# Cycling kinematics in healthy adults for musculoskeletal rehabilitation guidance

**DOI:** 10.1186/s12891-021-04905-2

**Published:** 2021-12-15

**Authors:** Haeun Yum, Hyang Kim, Taeyong Lee, Moon Seok Park, Seung Yeol Lee

**Affiliations:** 1 Division of Mechanical and Biomedical Engineering, Ewha Womans University, Seoul, South Korea; 2 New Horizon Biomedical Engineering Institute, Myongji Hospital, Goyang, Gyeonggi-do South Korea; 3 Graduate Program in System Health Science and Engineering (BK21 Plus Program), Ewha Womans University, Seoul, South Korea; 4 Department of Orthopaedic Surgery, Seoul National University Bundang Hospital, Seongnam, Gyeonggi-do South Korea; 5 Department of Orthopaedic Surgery, Myongji Hospital, Hanyang University College of Medicine, 55, Hwasu-ro 14beon-gil, Deogyang-gu, Goyang-si, Gyeonggi-do 10475 South Korea

**Keywords:** Cycling kinematics, Musculoskeletal rehabilitation, Range of motion

## Abstract

**Background:**

Stationary cycling is commonly used for postoperative rehabilitation of physical disabilities; however, few studies have focused on the three-dimensional (3D) kinematics of rehabilitation. This study aimed to elucidate the three-dimensional lower limb kinematics of people with healthy musculoskeletal function and the effect of sex and age on kinematics using a controlled bicycle configuration.

**Methods:**

Thirty-one healthy adults participated in the study. The position of the stationary cycle was standardized using the LeMond method by setting the saddle height to 85.5% of the participant’s inseam. The participants maintained a pedaling rate of 10–12 km/h, and the average value of three successive cycles of the right leg was used for analysis. The pelvis, hip, knee, and ankle joint motions during cycling were evaluated in the sagittal, coronal, and transverse planes. Kinematic data were normalized to 0–100% of the cycling cycle. The Kolmogorov-Smirnov test, Mann-Whitney U test, Kruskal-Wallis test, and k-fold cross-validation were used to analyze the data.

**Results:**

In the sagittal plane, the cycling ranges of motion (ROMs) were 1.6° (pelvis), 43.9° (hip), 75.2° (knee), and 26.9° (ankle). The coronal plane movement was observed in all joints, and the specific ROMs were 6.6° (knee) and 5.8° (ankle). There was significant internal and external rotation of the hip (ROM: 11.6°), knee (ROM: 6.6°), and ankle (ROM: 10.3°) during cycling. There was no difference in kinematic data of the pelvis, hip, knee, and ankle between the sexes (*p* = 0.12 to 0.95) and between different age groups (*p* = 0.11 to 0.96) in all anatomical planes.

**Conclusions:**

The kinematic results support the view that cycling is highly beneficial for comprehensive musculoskeletal rehabilitation. These results might help clinicians set a target of recovery ROM based on healthy and non-elite individuals and issue suitable guidelines to patients.

**Supplementary Information:**

The online version contains supplementary material available at 10.1186/s12891-021-04905-2.

## Background

Musculoskeletal rehabilitation programs aim at comprehensive orthopedic rehabilitation post-surgery or injury. Rehabilitation helps patients regain muscle and joint function and restores bone health by building strength and restoring flexibility and mobility, which reduces pain. The bone strength and function of the joints declines with aging [1]; moreover, many countries have a high proportion of an aging population. Thus, musculoskeletal rehabilitation is of considerable importance.

Stationary cycling is commonly recommended for individuals with various disabilities, such as knee osteoarthritis and compromised function of the joints following surgeries like anterior cruciate ligament (ACL) reconstruction and total hip arthroplasty. Cycling reduces the load on the knee joint [2, 3] and ACL [4–7]; the tibiofemoral compressive forces during cycling are between 0.3 and 2 times the body weight, while other full weight-bearing rehabilitative exercises (e.g., walking, stair ascent/descent) generate forces of approximately 2–4 times the body weight [4, 8–10]. The patellofemoral compressive force [9, 11], shear stress [12], tibiofemoral shear force [13, 14], and ACL strain [9, 11, 15, 16] are low during cycling; nevertheless, the quadriceps and hamstring muscles are strengthened as the knee stability increases [7, 12, 17–19]. Pedaling also increases the range of motion (ROM) of the hip, knee, and ankle joints [17, 20–23].

Many studies have investigated joint kinematics during cycling; however, most have been conducted on patients with orthopedic disabilities [12, 15, 24]. Few studies have investigated joint kinematics in healthy individuals. Furthermore, when healthy subjects were included in some of the studies, they were professional or experienced cyclists [25–28], and the study did not focus on target ROMs for rehabilitation purposes. Additionally, most studies analyzed the two-dimensional kinematic data and focused on the sagittal plane joint kinematics during cycling [1]. The three-dimensional (3D) kinematic data from individuals with healthy musculoskeletal function can serve as a clinical guide for appropriate cycling interventions, leading to more consistent results of the rehabilitation program. Therefore, this study aimed to elucidate 3D kinematics of the lower extremity joints in individuals with healthy musculoskeletal function and non-elite adults during cycling, and determine the kinematic differences by sex and age, and provide the literature on cycling rehabilitation.

## Methods

This prospective study was approved by the institutional board of our hospital. Informed consent was obtained from all participants. All procedures were performed in accordance with the relevant guidelines.

We competitively recruited individuals aged > 18 years (i.e., legal adults) through public notice or advertisement. Patients diagnosed with any musculoskeletal disease, those with a history of musculoskeletal trauma (including ligamentous injury and fracture), and those who were diagnosed with musculoskeletal deformities after the physical examination were excluded from the study. The participants were categorized into different age groups to determine the effects of age on kinematics. The categorization was group 1: < 20 years old, group 2: 21-35 years old, group 3: 36-50 years old, and group 4: > 50 years old. The physical examination was performed by a surgeon with 14 years of experience in orthopedic practice.

### Measuring the kinematic data

**Fig. 1:**
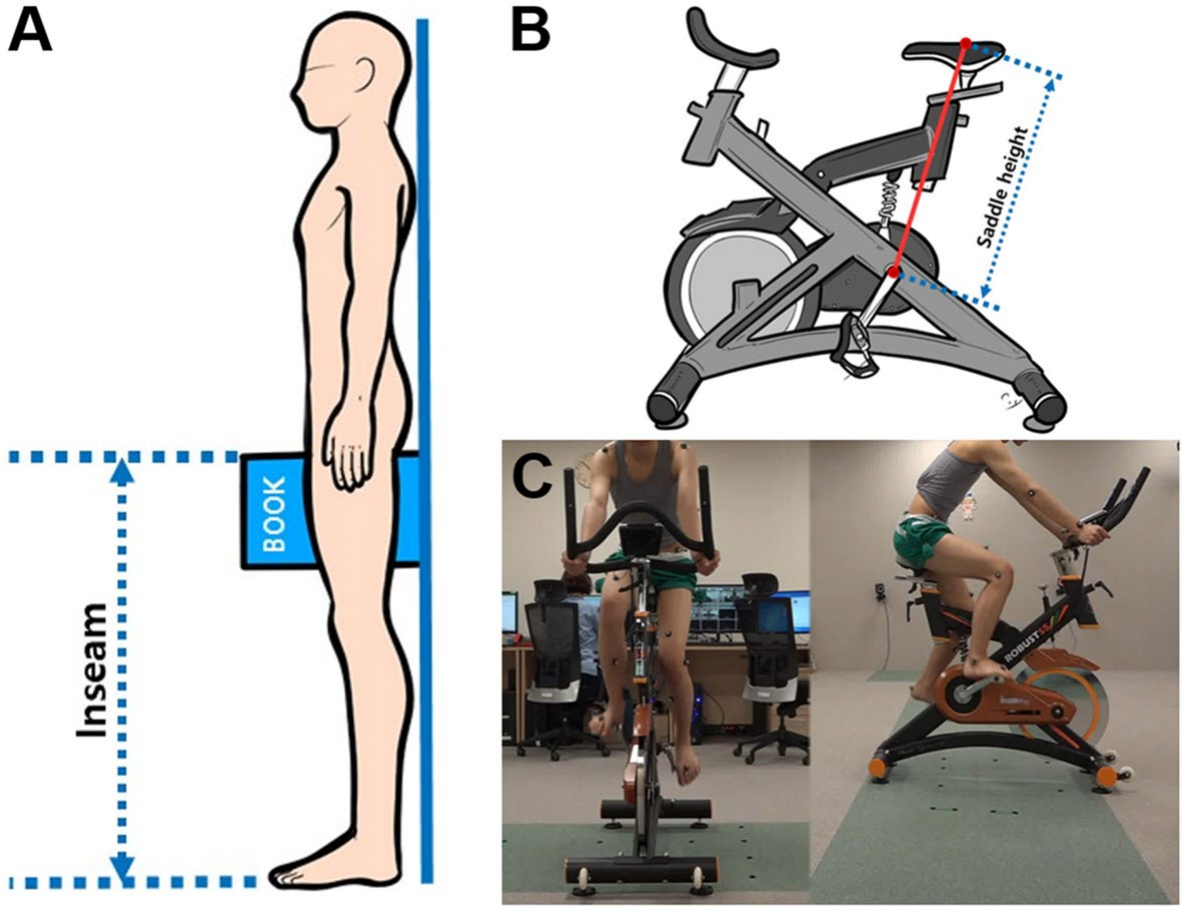
Measurements for the inseam (**A**) and saddle height (**B**), and the experimental setup showing a participant cycling on the stationary bike with the Helen Hayes marker set attached (**C**)

The bicycle settings can affect cycling performance. Thus, the position of each participant on the stationary cycle was standardized using the LeMond method, which is widely used and based on the empirical experience of the famous cyclist, Greg LeMond [29, 30]. The saddle height was measured from the center of the bottom bracket to the top of the seat along the seat tube. The saddle height was set at 88.3% of the distance between the highest point of one’s inner thigh to the heel of one’s foot, called an inseam (Fig. 1). This percentage is based on the average height of Westerners, and it is common to multiply the length by 0.855 for application to Asians [31]. Therefore, the configuration of the saddle height was set at 85.5% of the inseam in this study.

A pedaling rate of 10–12 km/h was required throughout the test. To ensure that the rate was maintained, participants rode a bicycle ergometer (HealthWay, ROBUST S5, Korea) for several minutes before recording the kinematics. Data collection was started when the participants were acquainted with the speed and maintained a steady rate. We obtained the 3D kinematic data using a Kestrel Digital System (Motion Analysis, Rohnert Park, CA, USA) equipped with 10 cameras. The Helen Hayes marker set was used to place markers on the top, back, and front of the head; bilateral acromion and olecranon processes and both wrists, anterior superior iliac spine (ASIS), thigh wand, lateral epicondyle of femur, shank wand, lateral malleolus, posterior surface of calcaneus; space between the second and third metatarsal heads, and sacrum [32]. Three additional markers were attached. One was placed on the back for offset, and the remaining two markers were attached on both sides of the sacral marker to create markers in case the ASIS marker disappeared when riding the bike. The movements of the pelvis, hip, knee, and ankle joints during cycling were evaluated in the sagittal, coronal, and transverse planes. The kinematic data were normalized to 0–100% of the cycling session. The right pedal at 0° was designated as 0%, and the right pedal after one 360° rotation (i.e., returned to 0°) was designated as 100%. Based on clinical gait analysis studies that involve more heterogeneous motions than those when cycling, the average value of three successive cycles in the middle of the trail was used for analysis [33].

### Statistical analyses

The lower extremity kinematics for the participants’ right side were used for analysis to avoid duplication of demographics [34]. The Kolmogorov-Smirnov test was used to verify the normality of the distribution of continuous variables. Descriptive statistics (i.e., mean ± standard deviation) were used to summarize the participants’ demographic and kinematic data. Comparisons between male and female groups were made using the Mann-Whitney U test based on data characteristics. The Kruskal-Wallis test was used to compare the kinematics between age groups. K-fold cross-validation was used to evaluate machine-learning models with a limited data sample. All statistical analyses were conducted using SPSS version 20.0 (IBM Co., Chicago, IL), and a *p*-value < 0.05 was considered significant.

## Results

Thirty-one participants were finally included in this study. The mean age at the time of examination was 35.0 ± 13.8 years (range, 18.2–68.1 years) (Table 1, Supplement 1). The mean saddle height was 68.0 ± 1.7 cm (range, 64.9–72.2 cm) for men and 64.8 ± 1.1 cm (range, 63.4–66.2 cm) for women (Table 1).

**Table 1:** Patient demographics

Parameter	Value
No. of subjects (Male/Female)	31 (23/8)
Age	35.0 ± 13.8 (18.2 – 68.1)
Height	172.1 ± 5.1 (162.9 – 185.0)
Male	174.1 ± 4.2 (166.8 – 185.0)
Female	166.5 ± 2.9 (162.9 – 170.0)
Inseam	78.5 ± 2.4 (74.2 – 84.5)
Male	79.5 ± 1.9 (75.9 – 84.5)
Female	75.8 ± 1.3 (74.2 – 77.4)
Saddle height	67.1 ± 2.1 (63.4 – 72.2)
Male	68.0 ± 1.7 (64.9 – 72.2)
Female	64.8 ± 1.1 (63.4 – 66.2)

Age, Height, Inseam, and Saddle height; mean ± standard deviation (range)

Age = decimal years

Saddle height = inseam × 0.855

**Fig. 2:**
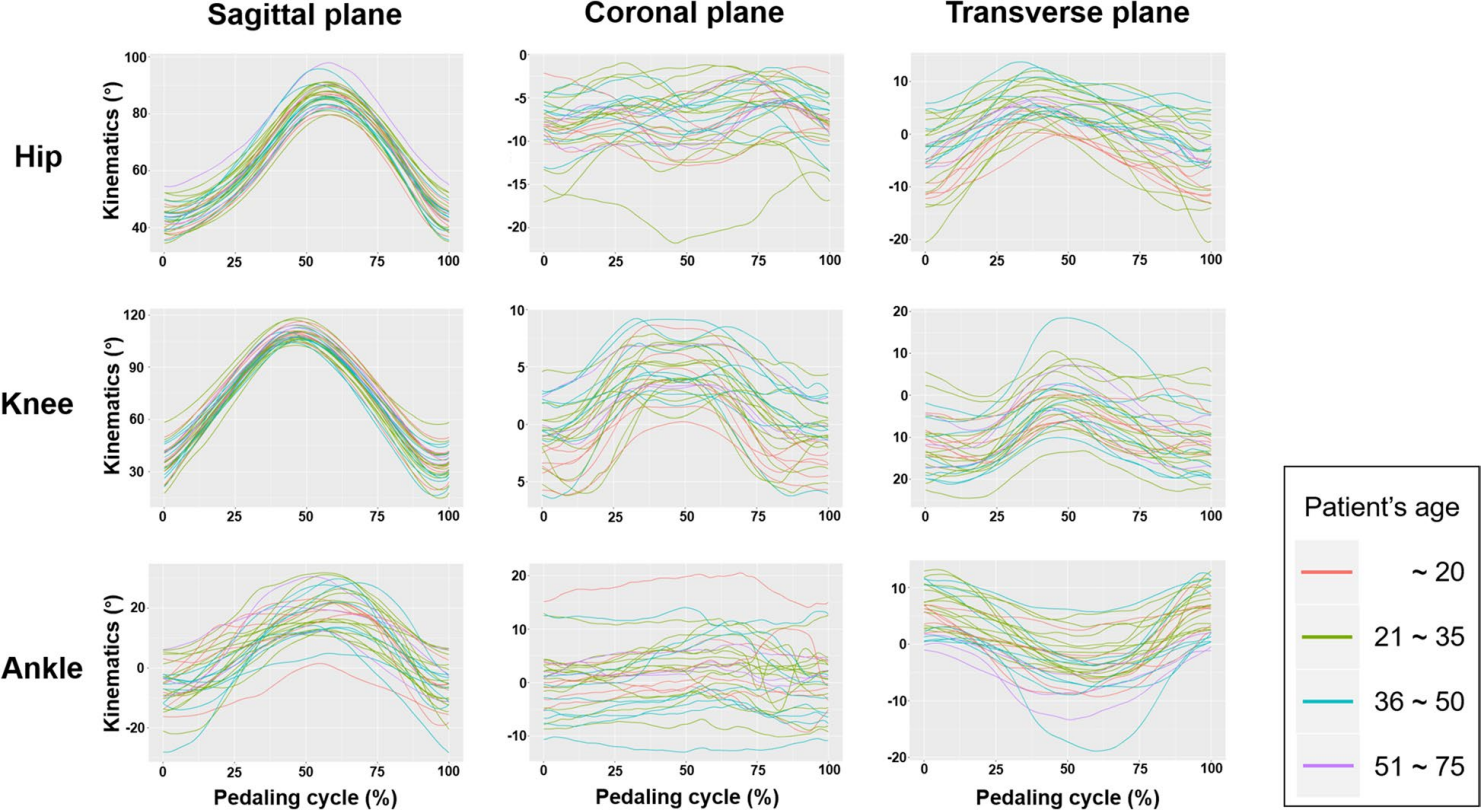
Hip, knee, and ankle joint angles for one complete revolution (0°–360°) of the bicycle crank for each participant’s right leg in the sagittal, coronal, and transverse planes

The joint motions were observed in all three planes (Table 2, Fig. 2). During cycling, the pelvis moved very little in the sagittal plane. The ROMs were 43.9 ± 3.7° (hip), 75.2 ± 7.2° (knee), and 26.9 ± 10.5° (ankle). The movement in the coronal plane was observed in all joints; particularly, the knee ROM was 6.6 ± 2.7°, and the ankle ROM was 5.8 ± 3.2°. The movement in the transverse plane was also observed in all the major joints of the lower extremity. Internal and external rotation occurred in the hip (11.6 ± 4.5°), ankle (10.3 ± 4.9°), and mainly in the knee joints (6.6 ± 2.7°).

**Table 2:** Sagittal, coronal, and transverse plane kinematics of the lower extremity during cycling

		Range of motion (•)		Maximum value (•)
Sagittal plane	Pelvis	1.6 ± 0.6 (0.7 – 3.6)	Posterior tilt	−15.0 ± 3.5 (− 24.1 – − 7.7)
			Anterior tilt	16.6 ± 3.6 (8.9 – 25.3)
	Hip	43.9 ± 3.7 (36.7 – 51.5)	Extension	−43.0 ± 5.1 (− 54.4 – − 34.5)
			Flexion	86.9 ± 4.3 (79.7 – 98.0)
	Knee	75.2 ± 7.2 (60.1 – 94.1)	Extension	−34.0 ± 9.8 (− 57.2 – − 14.7)
			Flexion	109.3 ± 3.9 (102.7 – 118.5)
	Ankle	26.9 ± 10.5 (10.8 – 47.0)	Dorsiflexion	7.6 ± 8.1 (− 6.2 – 28.3)
			Plantar flexion	19.2 ± 7.6 (1.6 – 31.8)
Coronal plane	Pelvis	7.1 ± 2.5 (2.0 – 11.7)	Rt side up	3.5 ± 2.1 (−1.2 – 7.2)
			Rt side down	3.6 ± 2.4 (−1.1 – 7.8)
	Hip	5.0 ± 1.8 (1.6 – 10.8)	Adduction	10 ± 3.4 (5.9 – 21.8)
			Abduction	−5.1 ± 2.9 (−13.6 – −0.9)
	Knee	6.6 ± 2.7 (2.5 – 12.0)	Varus	1.6 ± 2.8 (−4.2 – 6.44)
			Valgus	5.0 ± 2.2 (0.3 – 9.2)
	Ankle	5.8 ± 3.2 (2.1 – 14.2)	Inversion	1.5 ± 6.0 (−14.1 – 13.0)
			Eversion	4.3 ± 6.5 (−10.2 – 20.6)
Transverse plane	Pelvis	3.2 ± 1.9 (0.9 – 8.7)	Internal ROT	2.4 ± 3.1 (−4.2 – 8.8)
			External ROT	0.8 ± 3.3 (−4.3 – 12.5)
	Hip	11.6 ± 4.5 (3.3 – 23.8)	Internal ROT	6.3 ± 3.5 (−0.0 – 13.7)
			External ROT	5.3 ± 6.1 (−5.9 – 20.6)
	Knee	6.6 ± 2.7 (2.5 – 12.0)	Internal ROT	5.0 ± 2.2 (0.3 – 9.2)
			External ROT	1.6 ± 2.8 (−4.2 – 6.4)
	Ankle	10.3 ± 4.9 (3.9 – 22.0)	Internal ROT	4.4 ± 5.1 (−5.7 – 18.9)
			External ROT	6.0 ± 4.3 (−1.0 – 13.2)

*Rt* Right, *ROT* Rotation

The kinematic data of the pelvis, hip, knee, and ankle joints did not differ between the sexes (*p* = 0.12 to 0.95) or between the different age groups (*p* = 0.11 to 0.96) in all anatomical planes. In k-fold cross-validation of the age groups, the area under the curve was between 0.475 and 0.610.

## Discussion

Cycling is one of the most effective orthopedic rehabilitation methods to recover joint ROM with less weight load. Most studies have examined cycling in two dimensions, especially the sagittal plane [21, 35, 36]; however, recent studies have indicated that movements in the coronal and transverse also occur during cycling [1, 11, 27]. In recent times, 3D motion analysis is becoming critical for assessing the full rehabilitation potential. Thus, this study evaluated the 3D lower limb kinematics of people with healthy musculoskeletal function during stationary cycling and aimed to provide guidance for a target recovery ROM for physical rehabilitation. This study showed that considerable movement occurs in the sagittal plane and in the coronal and transverse planes. We also found that customizing the saddle height leads to constant joint kinematics.

The saddle position is often selected based on comfort. An improper position can lead to knee joint overuse injuries [2, 35, 37–39] and inconsistent kinematics. Numerous methods have been proposed to determine the appropriate saddle height configuration [11]. We selected the LeMond method since it is common, reliable [37], and simple to apply, which is important because most patients use public bicycles in clinics for rehabilitation purposes rather than personal bicycles. We adjusted the saddle height to the length of each participant’s inseam, considering the Asian-specific multiplication ratio. To our knowledge, this is the first study that considered the cycling rehabilitation environment based on race and individuals.

**Fig. 3:**
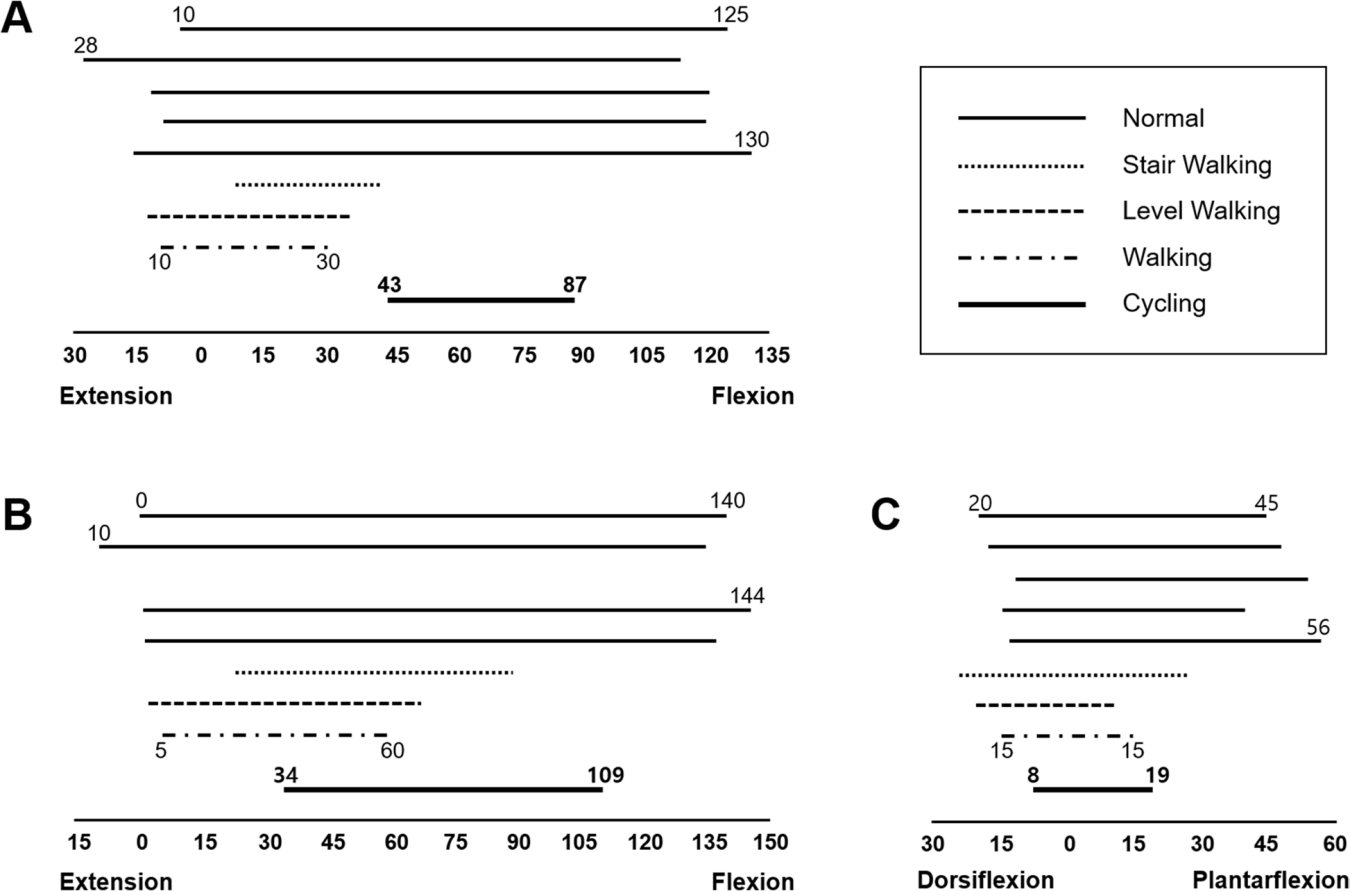
Comparisons between the normal range of motions (ROM data reported by Kendall et al. [43, 44], Ericson et al. [7], the American Academy of Orthopedic Surgeons [40], Boone et al. [41], Roaas et al. [42], and Soucie et al. [45]), and joint excursion during walking (Neumann [47] and Pietraszewski et al. [46]), stair or level walking (Ericson et al. [7]), and cycling (present study) of hip (**A**), knee (**B**), and ankle (**C**) joints. (degrees)

We found sagittal, coronal, and transverse movements in all joints during standardized ergometer cycling, enabling comprehensive rehabilitation guidance. When the sagittal joint ROMs obtained during ergometer cycling were compared with the mean values of normal ROMs, the hip ROM was approximately 31%, the knee ROM was approximately 54%, and the ankle ROM was approximately 42% of normal [7, 40–45] (Fig. 3). The cycling kinematics of the lower limb joints were also compared to normal walking kinematics. The normal sagittal plane ROM during a human gait cycle is approximately 45° in the hip (ranging from 10° [extension] to 35° [flexion]), 55° in the knee (ranging from 5° [flexion] to 60° [flexion]), and 30° in the ankle (ranging from 15° [dorsiflexion] to 15° [plantarflexion]) [46, 47]. This indicates that the hip and knee joints were much more flexed during cycling and that the ankle joint motion was similar to that during walking. The overall joint motion during pedaling might not have an advantage over that during walking. However, the range of angles in which the joint motion occurred during cycling was different from that during walking; this suggests that pedaling has effects that cannot be achieved only by walking. Thus, in terms of kinematics, cycling for musculoskeletal rehabilitation is highly recommended. Cycling could recover a partial ROM, and additional rehabilitative exercise is necessary to restore the ROM that cannot be recovered by cycling. In addition to walking, various other exercises could be included and cycling could be one. Lower limb kinematics are influenced by the saddle height [7, 11, 26]. Thus, the hip, knee, and ankle joint motions can be adjusted by changing the saddle height. For example, more plantar flexion can be achieved by increasing the saddle height. Further investigations are necessary to determine how cycling ROMs could be broadened for rehabilitation.

For the knee and ankle joints, significant internal and external rotation and coronal plane movements were observed. Cycling is recommended for individuals with knee disabilities because it exerts less weight on the knee [4]. However, cycling considerably rotates the knee, which should be considered before initiating bicycle rehabilitation for injuries adversely affected by rotational motion, such as meniscus and ankle ligament injuries. Understanding the 3D joint kinematics in healthy and normal individuals might help clinicians plan a target recovery ROM and issue guidance to patients.

This study attempted to recruit adult patients across all age groups for comparison according to sex and 10-year age groups. Pelvis, hip, knee, and ankle joint kinematics did not differ between the sexes or between age groups, indicating that customizing the saddle height per individual results in constant kinematics regardless of sex and age. No studies have investigated the effects of sex and age on joint kinematics in the context of rehabilitation. Thus, these results provide a good reference for planning lower limb rehabilitation.

There were a few limitations in this study. First, there were a small number of participants. This was a pilot study in preparation for a large sample size study, and to overcome the small sample size, additional k-fold cross-validation was conducted. Second, the saddle height was set considering an empirical percentage for leg length (0.855 for Asians), and the bicycle configuration did not consider the handlebar position. Comparative research is necessary to determine whether other bicycle configurations have an effect on joint ROM or rehabilitation.

## Conclusions

This study found that stationary cycling generates movement in the sagittal, coronal, and transverse planes, facilitating comprehensive lower limb rehabilitation. The findings can serve as a guide for setting the target kinematics during musculoskeletal rehabilitation with a stationary bicycle for individuals with orthopedic disabilities. The saddle height was adjusted for each participant; this led to consistent joint motions. Given the limited number of studies on bicycle 3D movement among the general population (non-professional athletes), further work is warranted to determine a suitable ROM for cycling rehabilitation customizable according to the race and physical conditions.

## Supplementary Information

**Additional file 1: Supplement 1**. Subjects’ data.

## Data Availability

The datasets used and/or analyzed during this study are available from the corresponding author on reasonable request.

## Abbreviations

3D: Three-dimensional
ROM: Range of motion
ACL: Anterior cruciate ligament
Rt: Right
ROT: Rotation
