# Supplementary material for: Cycling kinematics in healthy adults for musculoskeletal rehabilitation guidance
Source: BMC Musculoskelet Disord. 2021 Dec 15;22:1044. doi: 10.1186/s12891-021-04905-2 (PMC8675512; doi:10.1186/s12891-021-04905-2)
Supplement: Supplementary file 1 — Additional file 1: Supplement 1. Subjects’ data. [file 12891_2021_4905_MOESM1_ESM.docx]

| Supplement 1. Subjects’ data | | | | | |
| --- | --- | --- | --- | --- | --- |
| No. | Sex | Age | Height | Inseam | Saddle height |
| 1 | F | 32.3 | 162.9 | 74.2 | 63.4 |
| 2 | M | 26.4 | 176.4 | 80.4 | 68.7 |
| 3 | M | 24.1 | 171.0 | 78.1 | 66.8 |
| 4 | M | 36.8 | 177.0 | 80.9 | 69.2 |
| 5 | F | 47.2 | 163.0 | 74.2 | 63.4 |
| 6 | M | 18.3 | 166.8 | 75.9 | 64.9 |
| 7 | M | 35.7 | 176.0 | 80.4 | 68.7 |
| 8 | F | 19.8 | 164.7 | 75.1 | 64.2 |
| 9 | M | 18.9 | 175.1 | 80.0 | 68.4 |
| 10 | M | 23.8 | 172.0 | 78.6 | 67.2 |
| 11 | M | 68.2 | 170.6 | 77.7 | 66.4 |
| 12 | F | 25.7 | 169.0 | 76.9 | 65.7 |
| 13 | F | 19.8 | 170.0 | 77.4 | 66.2 |
| 14 | F | 20.4 | 168.0 | 76.4 | 65.3 |
| 15 | M | 36.4 | 174.0 | 79.5 | 68.0 |
| 16 | F | 30.3 | 165.0 | 75.1 | 64.2 |
| 17 | M | 26.2 | 176.0 | 80.4 | 68.7 |
| 18 | M | 34.7 | 178.3 | 81.3 | 69.5 |
| 19 | M | 24.9 | 175.0 | 80.0 | 68.4 |
| 20 | M | 45.7 | 175.0 | 80.0 | 68.4 |
| 21 | M | 31.8 | 168.0 | 76.8 | 65.7 |
| 22 | F | 29.1 | 169.0 | 76.9 | 65.7 |
| 23 | M | 48.8 | 173.0 | 79.1 | 67.6 |
| 24 | M | 51.8 | 170.0 | 77.7 | 66.4 |
| 25 | M | 38.9 | 167.0 | 76.3 | 65.2 |
| 26 | M | 39.8 | 179.0 | 81.8 | 69.9 |
| 27 | M | 47.1 | 185.0 | 84.5 | 72.2 |
| 28 | M | 39.7 | 173.0 | 79.1 | 67.6 |
| 29 | M | 56.9 | 172.0 | 78.6 | 67.2 |
| 30 | M | 19.8 | 175.0 | 80.0 | 68.4 |
| 31 | M | 67.6 | 178.0 | 81.3 | 69.5 |
| Age = decimal years  Height, Inseam, and Saddle height = cm | | | | | |
